# High Temperature and Power Dependent Photoluminescence Analysis on Commercial Lighting and Display LED Materials for Future Power Electronic Modules

**DOI:** 10.1038/s41598-019-52126-4

**Published:** 2019-11-14

**Authors:** Abbas Sabbar, Syam Madhusoodhanan, Sattar Al-Kabi, Binzhong Dong, Jiangbo Wang, Stanley Atcitty, Robert Kaplar, Ding Ding, Alan Mantooth, Shui-Qing Yu, Zhong Chen

**Affiliations:** 1 Department of Electrical Engineering, University of Arkansas, Fayetteville, Arkansas 72701 USA; 2 HC SemiTek (Suzhou), 28 Chenfeng Road, Zhangjiagang, Jiangsu, 215600 P.R. China; 3 Sandia National Laboratories, Albuquerque, New Mexico 87185 USA; 4 School of Electrical, Computer and Energy Engineering, Arizona State University, Phoenix, Arizona USA

**Keywords:** Electronic devices, Photonic devices

## Abstract

Commercial light emitting diode (LED) materials - blue (i.e., InGaN/GaN multiple quantum wells (MQWs) for display and lighting), green (i.e., InGaN/GaN MQWs for display), and red (i.e., Al_0.05_Ga_0.45_In_0.5_P/Al_0.4_Ga_0.1_In_0.5_P for display) are evaluated in range of temperature (77–800) K for future applications in high density power electronic modules. The spontaneous emission quantum efficiency (QE) of blue, green, and red LED materials with different wavelengths was calculated using photoluminescence (PL) spectroscopy. The spontaneous emission QE was obtained based on a known model so-called the ABC model. This model has been recently used extensively to calculate the internal quantum efficiency and its droop in the III-nitride LED. At 800 K, the spontaneous emission quantum efficiencies are around 40% for blue for lighting and blue for display LED materials, and it is about 44.5% for green for display LED materials. The spontaneous emission QE is approximately 30% for red for display LED material at 800 K. The advance reported in this paper evidences the possibility of improving high temperature optocouplers with an operating temperature of 500 K and above.

## Introduction

The need for high-temperature optocouplers, which can be reliably operated over 500 K, is currently driven by the high-temperature requirements in the next generation high-density power electronics^1^. The optoelectronic materials, which can be utilized at high temperatures, are essential for the advance of high temperature optocouplers. Therefore, the development of high temperature optocouplers, which can be operated at elevated temperatures (over 500 K) with an extended lifetime, requires a systematic study of optoelectronic materials and devices at high temperatures. There are numerous groups studied optoelectronic materials and devices from cryogenic temperature to room temperature^2–6^. However, systematic understandings on optoelectronic materials and devices at high temperatures (above 500 K) are still very limited^7–18^. We previously reported the spontaneous emission QE of one type of LED materials up to 800 K for the improvement of high temperature optocouplers^1^. The published material is indium gallium nitride (InGaN) based commercial green LED material for display applications. Power- and temperature-dependent photoluminescence (PL) measurements were carried out to estimate the changes in the spontaneous emission QE temperature ranging from 10 K to 800 K. The peak spontaneous emission QE of this green LED material for display is over 80% at 500 K while it is around 44% at 800 K. The so-called ABC model, which is the carrier recombination model, was used in the study to estimate the spontaneous emission QE. This model is typically used in temperature ranging from 10 K to 300 K. However, it was extended in our previous work up to 800 K to check the validity of the model at elevated temperatures.

In this paper, the study on the LED materials at high temperatures was expanded to commercial LED materials for both display and lighting applications. Commercially available LED displays generally utilize the combinations of RGB (Red, Green & Blue) LEDs to adjust the color and dimming level. Displays based on high brightness RGB LEDs can yield almost any color, as well as the white one. For the lighting application, the white light can be created by either the combinations of RGB LEDs or the phosphor induced blue LEDs^19^. By incorporating the phosphorus into the body of the blue LEDs, a portion of the blue light is changed to yellow. When blended with the yellow color, the blue LED will emit the white light. As shown in Table 1, we studied four types of commercial LED materials, which are - blue for display (i.e., sample A1), blue for lighting (i.e., sample A2), green for display (i.e., sample B) and red for display (i.e., sample C). The peak wavelength for A1, A2, B, and C are 467 nm, 448 nm, 515 nm, and 629 nm, respectively. All the blue and green LED materials are based on the InGaN/GaN MQWs on the sapphire substrates. The red LED material consists of the Al_0.05_Ga_0.45_In_0.5_P/Al_0.4_Ga_0.1_In_0.5_P MQWs on the GaAs substrate. All these commercial LED materials were systematically examined and compared to evaluate if they can satisfy the light output requirements in the optocouplers at high temperatures. The excitation pumping power in PL measurements was extended to a wide range of six orders of magnitudes (i.e., from 1 × 10^−4^ mW to 2.5 × 10^2^ mW). This paper explored the feasibility of these commercial display and lighting LED materials for high-temperature applications. This work serves as a foundation for advanced high-density power modules in which high temperature optocouplers as an isolation system.

**Table 1:** Commercial LEDs samples with different peak wavelength and compositions, which are characterized in this paper.

Application	Blue LED	Green LED	Red LED
Display	**A1** (467 nm) InGaN/GaN	**B** (515 nm) InGaN/GaN	**C** (629 nm) Al_0.05_Ga_0.45_In_0.5_P/Al_0.4_Ga_0.1_In_0.5_P
Lighting	**A2** (448 nm) InGaN/GaN		

## Results

**Figure 1:**
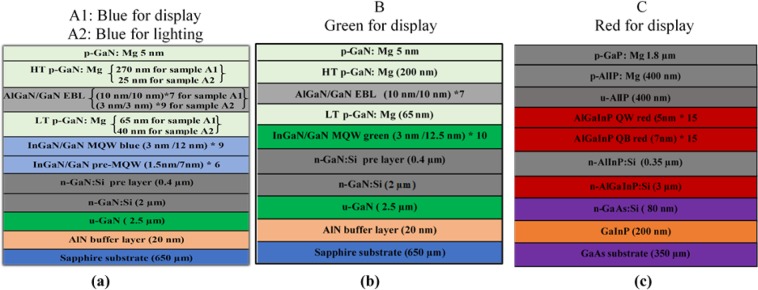
The Schematic epitaxial structure of four samples. **(a)** Sample A1 is the blue LED for display, and sample A2 is the blue LED for lighting. **(b)** Sample B is the green LED for display, and **(c)** Sample C is the red LED for display.

The schematic epitaxial structure of four commercial LED materials with different wavelengths, which are evaluated in this paper is shown in Fig. 1. Samples A1, A2, and B are the InGaN MQWs on the sapphire substrates. The difference between the blue for display and the blue for lighting is the thickness of, p-GaN (low and high temperature), and electron blocking layer (EBL) layers. The blue LED materials contain pre-quantum well and quantum well layers in their structures, whereas the green LED material contains only quantum well layers in their structure. Figure 1(c) illustrates the schematic epitaxial structure for the red LED material for display (sample C), which is based on the Al_0.05_Ga_0.45_In_0.5_P/Al_0.4_Ga_0.1_In_0.5_P MQWs on GaAs substrates. The detailed information of the LED epitaxy structures and their growth methods can be found described in the Method Section.

**Figure 2:**
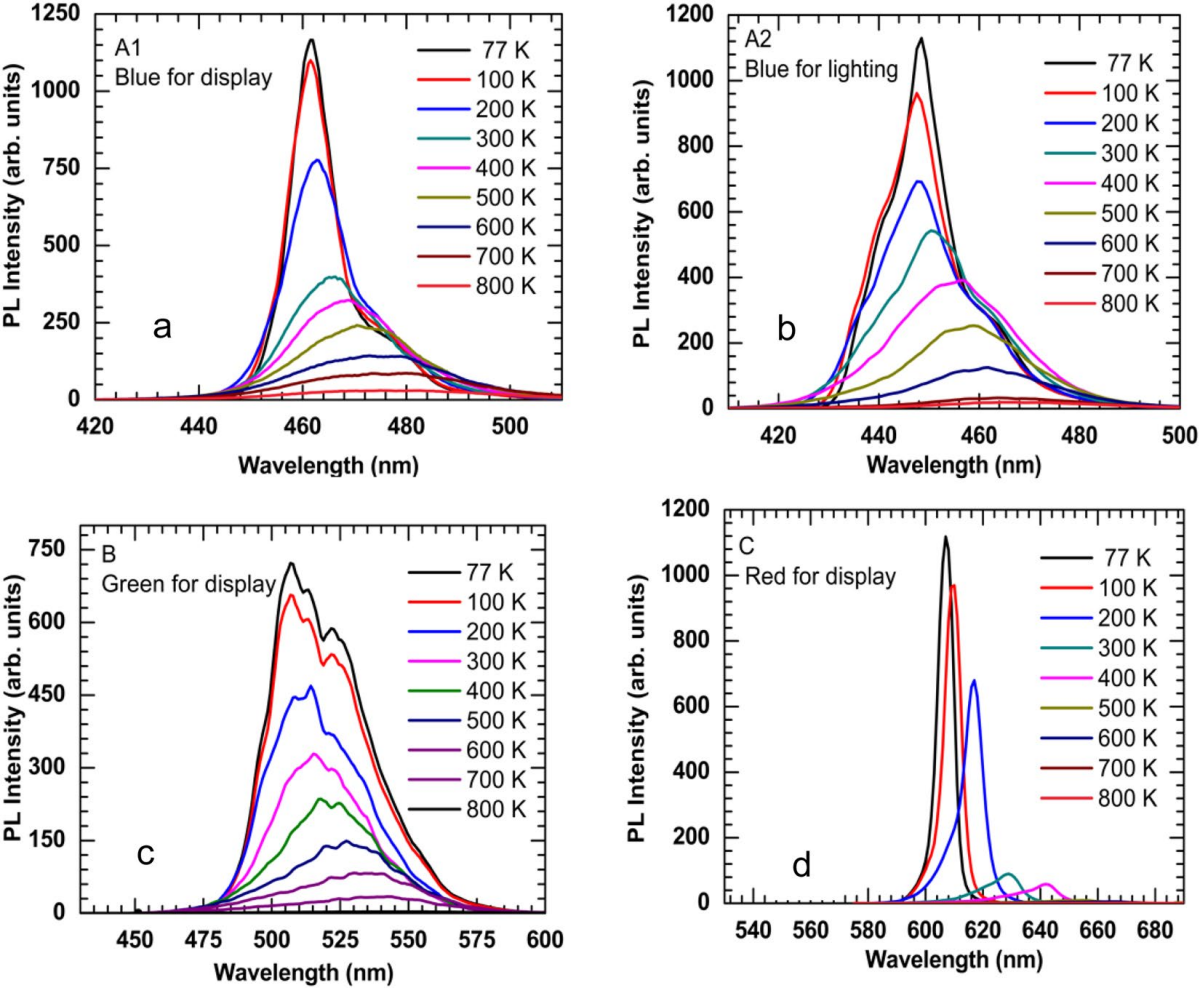
Temperature-dependent PL spectra of the samples from 77 K to 800 K using excitation power laser of 110 mW with an excitation wavelength of 395 nm for InGaN/GaN MQW samples and 532 nm for AlGaInP sample. (**a**) Sample A1 is the blue LED for display. (**b**) Sample A2 is the blue LED for lighting. (**c**) Sample B is the green LED for display, and (**d**) Sample C is the red LED for display.

Figure 2 shows the PL spectra for four different LED materials in temperature ranging from 77 K to 800 K. The excitation source for blue and green LED materials was a continuous-wave (CW) laser with an operational wavelength of 395 nm. For the red LED material, the excitation source was CW laser operated at 532 nm. The laser power was varied with a wide range of six orders of magnitudes (i.e., 1 × 10^−4^ mW- 2.5 × 10^2^ mW). The PL signals emitted from all samples are redshifted, which is as a result of the shrinking of the optical bandgap with elevating temperatures. The magnitude of redshift is increased with temperature. PL emission intensity increases with decreasing the temperatures. The multiple peaks in the spectrum of samples A1, A2, and B can be attributed to a Fabry-Perot cavity produced due to the interface between the AlN buffer layers and sapphire substrates^20^. In Fig. 2(a,b,c), the high energy region of the PL spectra, refereeing to the lower wavelength region, shows a change in intensity with respect to temperature. The lower energy region spectra tails, i.e., a higher wavelength region, merge indicating little dependence on the temperature. It was reported that the strong indium segregation in InGaN causes less temperature- dependent (almost independent) behavior of the low-energy region^21,22^. The indium segregation contributes to the creation of indium-rich regions like quantum-dot. The low-energy PL of the InGaN MQW samples is subjugated by the emission at localized states in the indium-rich regions. Also, it is worth to mention that the tail of the PL spectra on the high-energy region became broader as the temperature was increased due to the increasing number of the electron-hole pairs in the conduction and valance bands at elevated temperatures.

**Figure 3:**
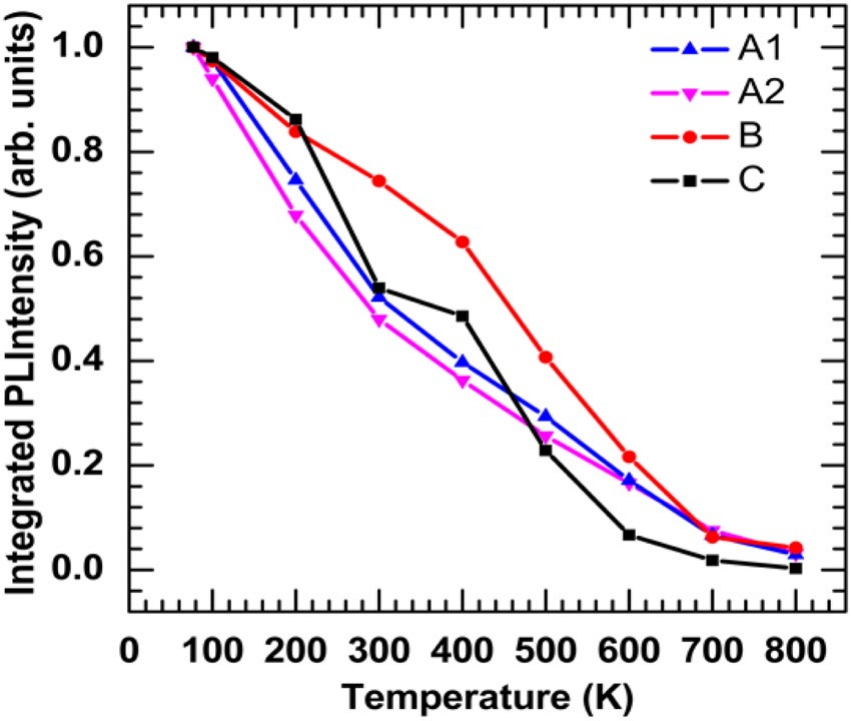
Normalized integrated photoluminescence intensities of InGaN/GaN and Al_0.05_Ga_0.45_In_0.5_P/Al_0.4_Ga_0.1_In_0.5_P MQWs are plotted as a function of temperatures using 110 mW as an excitation power.

**Figure 4:**
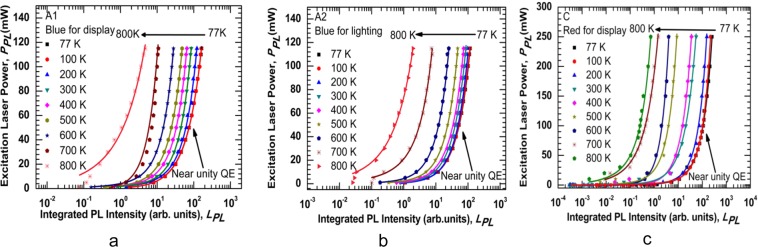
Excitation laser power is plotted against integrated PL of the samples from 77 K to 800 K to extract A_PL_, B_PL_, and C_PL_ coefficients, which are related to the (SRH), radiative, and Auger recombination processes, respectively using Eq. 1. (**a**) Sample A1 is the blue LED for display. (**b**) Sample A2 is the blue LED for lighting. (**c**) Sample C is the red LED for display.

The normalized temperature-dependent integrated PL spectra of four samples are shown in Fig. 3. It was observed that the total integrated PL intensity is reduced by one order of magnitude in temperature ranging from 77 K to 800 K. Elevating the temperature causes the reduction of luminescence because of the dominance of the non-radiative recombination at high temperatures. Since the injected electron-hole pairs have high thermal energy at higher temperatures, their confinement in the quantum wells decreases and eventually they escape the quantum wells and recombine non-radiatively, which leads the drop of the PL intensity. Sample B was reported in the previous work, and it is used in this work as a comparison with other samples^1^. The integrated PL intensity for samples A1, A2, and C as a function of the excitation laser power is plotted in Fig. 4(a,b,c), respectively. The experimental results are presented by different dote symbols, whereas the fits to Eq. 1 are presented by the solid lines. The recombination coefficients for the SRH, radiative, and Auger are represented by A_PL_, B_PL_, and C_PL,_ respectively_,_ were obtained from Fig. 4 to find the spontaneous emission QE. The tradition ABC model illustrates a good fit to the experimental data up to 800 K. As a result of the reduction of the radiative lifetime; the curves will move to the near unity QE curve at 77 K. As the temperature decreases, the experimental data will move to this curve due to an increasing B_PL_ coefficient. However, when the Auger recombination occurs as the dominant process (at high temperatures), the experimental data eventually move far from this curve. With the same injection levels, a notable reduction of the integrated PL intensity with elevating temperatures (particularly over 600 K) can be observed. As the temperature increases, the curvature of the curves declines, which indicates a drop in spontaneous emission QE at higher temperatures. This behavior could be attributed to the domination of the A_PL_ coefficient at higher temperatures.

**Figure 5:**
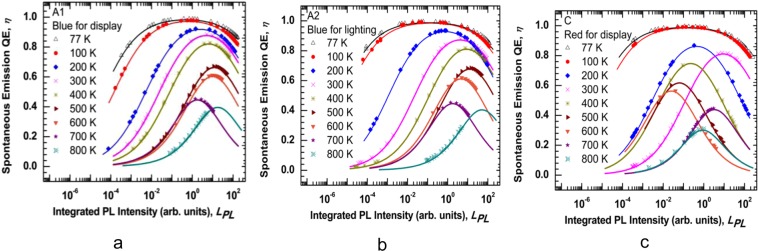
Spontaneous emission QE was extracted using the data fitting of Fig. 4 and Eq. 2. The spontaneous emission QE is plotted as a function of integrated PL intensity at different temperatures. (**a**) Sample A1 is the blue LED for display. (**b**) Sample A2 is the blue LED for lighting. (**c**) Sample C is the red LED for display.

The fit parameters extracted (A_PL_, B_PL_, and C_PL_) from Fig. 4 and used to calculate the spontaneous emission QE at different temperatures. The laser pumping power is plotted as a function of integrated PL intensity in Fig. 5(a,b,c) for samples A1, A2, and C, respectively. The details of the calculations are explained in the Method Section. The experimental results are represented by different dote symbols, whereas the fits to Eq. 2 are represented by the solid lines. The peak position of the spontaneous emission QE depends on the structure of the sample. From the peak position of the spectra, one can get the LED output intensity when it works with the highest efficiency for a particular temperature. The peak QE decreases as the temperature increase. However, the integrated PL intensity, at which peak QE occurs, increases initially and decreases after 500 K. Equation (1) is used to extract the laser pumping power from the integrated PL intensity at which the maximum QE occurs. A detailed study on the trend of QE change with respect to laser pumping power is explained in detail in the later part of the paper.

The curves in temperatures ranging from 77 K to 200 K show that the calculated data points are distributed on the entire curves. At this region, the radiative recombination is the dominant process. The curves in a range of temperatures from 300 K to 700 K show the calculated data are mainly at the left- hand and partially right- hand side of the peak spontaneous emission QE, which indicates the non-radiative processes to become the dominant process. At 800 K, the calculated data points for samples A1 and A2 do not reach the peak because the spontaneous emission QE peak requires the injected laser power to be above 110 mW, which is the upper limit of our current laser source. At this region, the calculated data points are located on the left-hand side of the peak spontaneous emission QE as an indication of the dominant of the C_PL_ coefficient in this region. This behavior refers to the domination of the non-radiative recombination process even at a low excitation laser power, when the temperatures are less than 300 K. At high temperatures (above 300 K), the domination of the non-radiative recombination process along with the leakage of thermally activated carriers from the QWs, cause the spontaneous emission QE to drop. From these results, it is concluded that the Auger and SRH recombination rates are dependable on the temperature- and injection-level.

**Figure 6:**
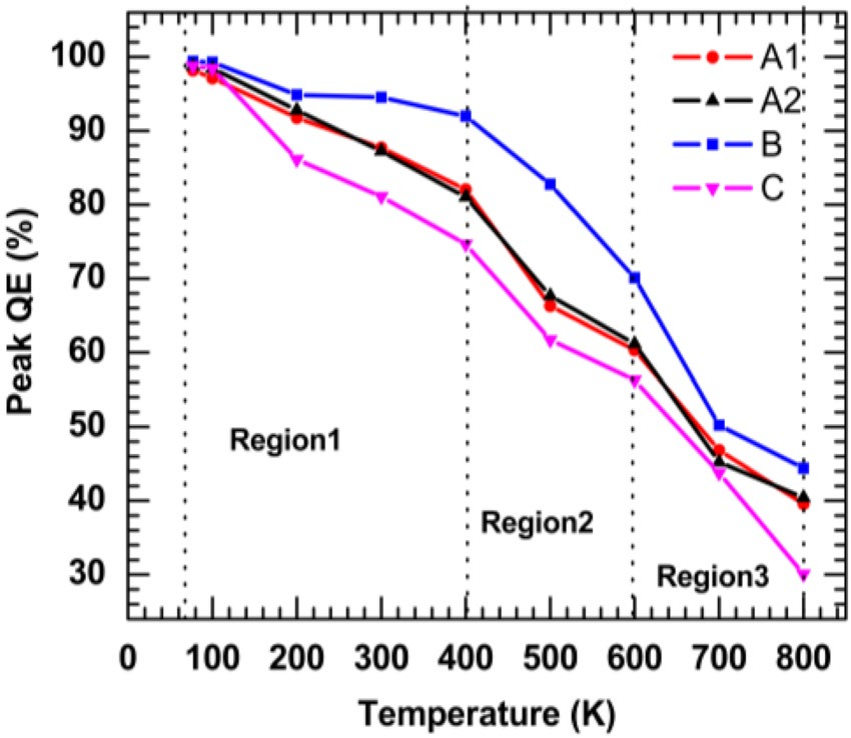
The peak of spontaneous emission QE, which was extracted from using Fig. 5, as a function of temperatures, was plotted for blue LED for display (A1), blue LED for lighting (A2), is green LED for display (B), and red LED for display (c) samples.

The temperature dependent spontaneous emission QE peak is plotted in Fig. 6. It can be seen that the peak of the spontaneous emission QE is almost unity (i.e., 99%) at 77 K, and it declines when the temperature is increased. Three regions can be observed from these results. Region 1 involves the data at a different temperature ranging from 77 K to 400 K. The peak of the spontaneous emission QE is over 80% for samples A1 and A2 while the spontaneous emission QE is over 90% for the sample B, which indicates the InGaN/GaN MQWs exhibits high thermal stability. Region 2 includes the data at a different temperature ranging from 400 K to 600 K. In this region, the peak of the spontaneous emission QE is still over 60% for sample A1 and A2, while the QE peak for sample B is 70%. Within the same region, sample C shows less spontaneous emission QE peak. Region 1 contains the data at a different temperature ranging from 600 K to 800 K where the peak of the spontaneous emission QE is 40–45% for InGaN/GaN MQWs (i.e., blue and green LED materials) and 30% for Al_0.05_Ga_0.45_In_0.5_P/Al_0.4_Ga_0.1_In_0.5_P MQWs (i.e., red LED material). This indicates that all these commercial LED materials for display and lighting applications have strong potential used for the future generation optoelectronic devices, which can be operated reliably at high temperatures over 500 K.

**Figure 7:**
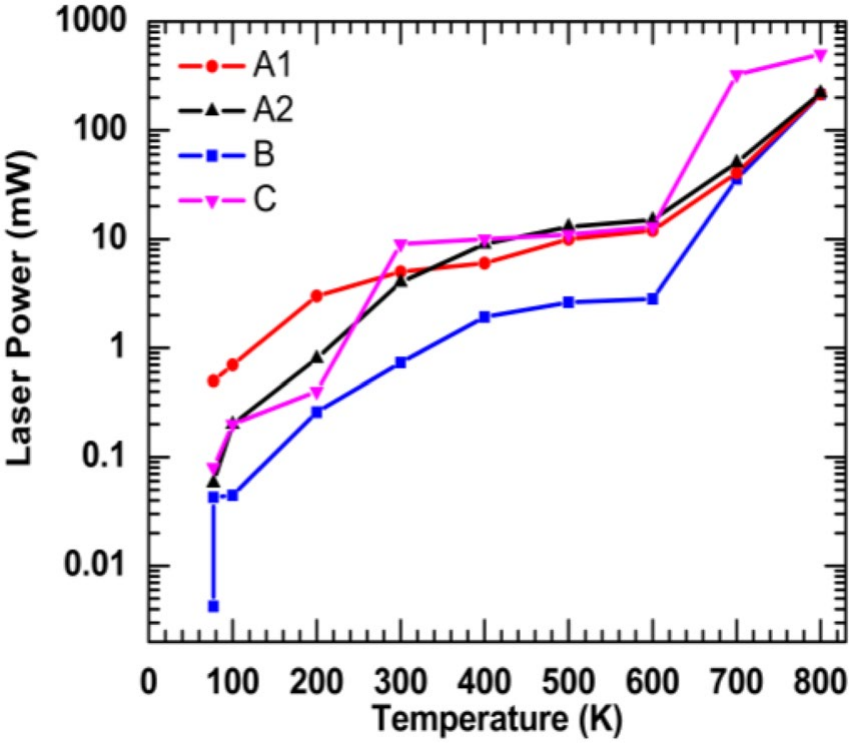
Excitation pump power for maximum quantum efficiency as a function of temperatures was plotted for the blue LED for display (A1), blue LED for lighting (A2), is the green LED for display (B), and the red LED for display (c) samples.

The temperature dependent laser power in which the maximum spontaneous emission QE of all commercial LED materials are obtained is plotted as a function of temperature in Fig. 7. The peak of spontaneous emission QE at temperatures below 600 K can be obtained with low injection levels (i.e., less than 20 mW). With increasing the temperature above 600 K, it is required to provide a comparatively high injection level to reach the maximum efficiency, as a result of the domination of the C_PL_ coefficient at high temperatures. The laser excitation power must be above 200 mW for samples A1, A2, and B to reach the peak spontaneous emission QE at 800 K. Sample C requires much higher injection levels at high temperature compared with other samples.

## Conclusion

In conclusion, commercially available epitaxial materials for display and lighting applications (i.e., InGaN/GaN and Al_0.05_Ga_0.45_In_0.5_P/Al_0.4_Ga_0.1_In_0.5_P MQWs) with different wavelengths were investigated up to 800 K using temperature- and power- PL measurements, which were carried out to estimate the spontaneous emission QE for these materials. As the temperature was increased from 77 K to 800 K, the integrated PL intensity was declined by one order of magnitude. The blue and green LED materials exhibit peaks QE of (40–44) % at 800 K while the red LED material show peak QE of 30% at 800 K. The peak of the spontaneous emission QE can be reached with less than 20 mW below 600 K for all four samples. These results prove the excellent potential for the commercial LEDs for display and lighting to be employed in the field of high-temperature optoelectronics since the samples show relatively high values of spontaneous emission QE at elevated temperatures.

## Method

Blue and green LED materials, which consist of InGaN/GaN MQW structures (i.e., sample A1, A2 and B), were all deposited on the sapphire (Al_2_O_3_) substrates. Sample B was described in detail in previous work. ^1^Before the growth, the sapphire substrate was cleaned. Then, deposit a 20-nm-thick AlN buffer layer followed by a 2.5-µm-thick undoped GaN layer and a 2-µm Si-doped n-GaN layer, respectively. For blue LEDs materials (samples A1 and A2), a pre-quantum well structure, which consists of six pairs of 1.5-nm undoped InGaN wells and 7-nm Si-doped GaN barriers, was grown on a 0.4-µm-thick n-GaN layer as shown in Fig. 1(a). Then, the MQW active region consists of nine periods of 3-nm-thick InGaN well layers and 12-nm-thick GaN barrier layers was formed. Next, a low-temperature Mg-doped p-GaN layer was deposited directly on the MQW, followed by a p-AlGaN layer (an electron blocking layer (EBL)). Lastly, a 5 nm thick p-GaN contact layer was grown as the last layer after high-temperature Mg-doped p-GaN layer was deposited on top of the EBL. For the green LEDs (sample B), there are no pre-quantum wells, as shown in Fig. 1(b). The MQW active region consists of ten pairs of 3-nm-thick InGaN well layers and 12.5-nm-thick GaN barrier layers.

Red LED material, which consists of Al_0.05_Ga_0.45_In_0.5_P/Al_0.4_Ga_0.1_In_0.5_P MQWs, was grown on the GaAs substrate (Fig. 1(c)). At first, the substrate was cleaned. Then, a 200-nm-thick GaInP etch stop layer was grown. Then, an 80 nm-thick Si-doped GaAs layer was deposited as an ohmic layer. After that, a 3-µm Si-doped n-AlGaInP layer was deposited followed by a 350-nm Si-doped n-AlInP layer. The quantum well structures are formed by fifteen pairs of 5-nm Al_0.05_Ga_0.45_In_0.5_P quantum well (QW) layers and 7-nm Al_0.4_Ga _0.1_In_0.5_P quantum barrier (QB) layers. After that, an undoped u-AlInP was deposited directly on the MQW. Finally, the Mg-doped p-AlInP layer was deposited followed by a 1.8-µm thick p-GaP contact layer.

The standard off-axis configuration was utilized to perform the temperature- and power-dependent PL measurements. For all blue and green LED materials, a continuous-wave laser functioned at 395 nm with a focused spot size of ~60 µm was used as an excitation source. For red LED material, also a continuous-wave laser functioned at 532 nm with a focused spot size of ~90 µm has used the excittion source. The power incident on the sample was wide-ranging from 1 × 10^−4^ mW to 2.5 × 10^2^ mW. The sample is mechanically retained to the sample holder, which was placed inside a cryostat. Multiple measurements were performed on the samples to ensure the characterization results are repeatable.

The calculations were discussed in detail previously^1,2^. The power law relation relating the integrated PL intensity of the spectrum and the excitation pumping power was used to calculate the spontaneous emission QE of the InGaN/GaN and AlGaInP/AlGaInP MQWs. The relation between the integrated PL intensity (L_PL_) and the excitation pumping power (P_PL)_ is given by

1 $${P}_{PL}={A}_{PL}{({L}_{PL})}^{1/2}+{B}_{PL}{L}_{PL}+{C}_{PL}\,{({L}_{PL})}^{3/2}$$

where A_PL_, B_PL_, and C_PL_ represente the recombination coefficients for the (SRH), radiative, and Auger recombination processes, respectively. These coefficients can be obtained by fitting Eq. (1) to the excitation pump power against the integrated PL intensity measurements. After the best fitting parameters extracted, the spontaneous emission quantum efficiency (η) can be calculated using Eq. 2

2 $$\eta ={(1+\frac{{A}_{PL}}{{B}_{PL}}(\frac{1-{\gamma }_{r}}{\sqrt{{L}_{PL}}})+\frac{{C}_{PL}}{{B}_{PL}}(1-{\gamma }_{r})\sqrt{{L}_{PL}})}^{-1}$$

The fraction of the photon emission reabsorbed by the active region is called the photon recycling factor, which is denoted by γ_r_ in Eq. 2. For this study, γ_r_ was assumed as an independent factor of laser injection levels. The excitation power-dependent PL measurements were used to obtain the spontaneous emission QE^1,2^ calculation using Eq. 2.
